# A Novel BD2-Selective Inhibitor of BRDs Mitigates ROS Production and OA Pathogenesis

**DOI:** 10.3390/antiox13080943

**Published:** 2024-08-02

**Authors:** Hyemi Lee, Jihye Choe, Min-Hee Son, In-Hyun Lee, Min Ju Lim, Jimin Jeon, Siyoung Yang

**Affiliations:** 1 Department of Biological Sciences, Sungkyunkwan University, Suwon 16419, Republic of Korea; hyemi0320@gmail.com; 2 Benobio Co., Ltd., Seongnam-si 13494, Republic of Korea; jihye.choe@benobio.com (J.C.); sonmh@benobio.com (M.-H.S.); bigwhale@benobio.com (I.-H.L.); 3 Department of Biomedical Sciences, Graduate School of Medicine, Ajou University, Suwon 16499, Republic of Korea; minju6021@ajou.ac.kr

**Keywords:** osteoarthritis, BBC0906, BET inhibitor

## Abstract

Bromodomain and extra-terminal domain (BET) family proteins regulate transcription and recognize lysine residues in histones. Selective BET inhibitors targeting one domain have attracted attention because they maintain normal physiological activities, whereas pan (nonselective) BET inhibitors do not. Osteoarthritis (OA) is a joint disorder characterized by cartilage degeneration for which no treatment currently exists. Here, we investigated whether the selective inhibition of BET proteins is an appropriate therapeutic strategy for OA. We focused on the development and characterization of 2-(4-(2-(dimethylamino)ethoxy)-3,5-dimethylphenyl)-5,7-dimethoxyquinazolin-4(3H)-one (BBC0906), a novel bromodomain 2 (BD2)-specific inhibitor designed to suppress OA progression. Using a DNA-encoded chemical library (DEL) screening approach, BBC0906 was identified because of its high affinity with the BD2 domain of BET proteins. BBC0906 effectively reduced reactive oxygen species (ROS) production and suppressed catabolic factor expression in chondrocytes in vitro. Moreover, in an OA mouse model induced by the destabilization of the medial meniscus (DMM), BBC0906 intra-articular injection attenuated cartilage degradation and alleviated OA. Importantly, BBC0906 selectively inhibits the BD2 domain, thus minimizing its potential side effects. We highlighted the therapeutic potential of targeting BET proteins to modulate oxidative stress and suppress cartilage degradation in OA. BBC0906 is a promising candidate for OA treatment, offering improved safety and efficacy.

## 1. Introduction

Osteoarthritis (OA) is a degenerative joint disorder characterized by increased articular cartilage degradation that results in articular dysfunction. Oxidative stress plays a critical role in the progression of OA [1]. Reactive oxygen species (ROS), such as superoxide, hydroxyl radicals, and hydrogen peroxide, are highly reactive molecules that induce aging and inflammation, leading to many diseases, including neurodegenerative disorders [2,3,4]. In joints with OA, dysregulated ROS production interrupts endogenous antioxidant defenses resulting in oxidative stress in chondrocytes, leading to the oxidation of biomolecules such as lipids, proteins, and nucleic acids [5,6,7].

Epigenetic mechanisms govern gene expression without altering the DNA sequences, as recently demonstrated [8,9,10]. Some of the most important epigenetic transcriptional regulators, bromodomain and extra-terminal (BET) proteins, belong to bromodomain-containing proteins (BRDs). The BET family of proteins, including bromodomain-containing 2 (BRD2), bromodomain-containing 3 (BRD3), bromodomain-containing 4 (BRD4), and bromodomain testis associated (BRDT), are representative molecules related to epigenetic mechanisms. The BET family commonly has two highly conserved tandem bromodomains, bromodomain 1 (BD1) and bromodomain 2 (BD2), and an extra-terminal structural domain. Two bromodomains with a four-helix bundle and two loops are located at the N-terminal end. The extracellular structure domain, which consists of a highly conserved sequence of approximately 80 amino acids, is located at the C-terminal end [11,12]. BET proteins recognize and bind to acetylated lysine residues, thereby regulating chromatin accessibility and gene transcription [13]. The BRD family of proteins participates in diverse cellular processes, including inflammation, cell proliferation, and differentiation, and their dysregulation is implicated in OA pathophysiology [14,15,16].

Moreover, the knockdown of BET proteins suppresses the expression of proinflammatory cytokine-induced catabolic factors [17]. This suggests that aberrant BRD protein expression affects OA pathogenesis. BET proteins have been highlighted as potential therapeutic targets for OA. However, the pathological relationship between BET proteins and OA remains poorly understood.

BRD proteins are involved in cellular ROS production. BRD4 inhibition decreases ROS production and increases cell viability under oxidative stress conditions [18]. In particular, JQ1, a BET inhibitor, blocks renal fibrosis and suppresses TGF-β-mediated oxidative stress [19]. Additionally, in diabetic rats, JQ1 enhances cognitive function by decreasing oxidative stress levels in the hippocampus [20].

The development of inhibitors that selectively target the BD2 domain of BET proteins has attracted considerable interest because of their potential therapeutic advantages. While broad BET inhibitors (BETi) targeting both the BD1 and BD2 domains have demonstrated efficacy in preclinical and clinical settings, particularly for cancer and inflammatory diseases, they can cause undesirable side effects, owing to the essential roles of BET proteins in normal cellular functions [21,22,23,24].

Recently, the selectivity of small-molecule compounds has improved, enabling the selective inhibition of specific proteins rather than inhibiting multiple proteins simultaneously. Therefore, the identification of BET inhibitors that selectively target one domain has become a research hotspot.

Therefore, 2-(4-(2-(dimethylamino)ethoxy)-3,5-dimethylphenyl)-5,7-dimethoxyquinazolin-4(3H)-one (BBC0906) has been developed as a BD2-specific inhibitor. BBC0906 reduces ROS production and mitogen-activated protein kinase (MAPK) signaling, leading to the reduced expression of catabolic factors that degrade cartilage. Moreover, in an animal model of OA, the intra-articular injection of BBC0906 inhibited the induction of OA and reduced the levels of ROS-related factors in joints.

## 2. Materials and Methods

### 2.1. Screening of the DNA-Encoded Chemical Library (DEL) Using the Bromodomains of BRD2, BRD3, and BRD4

Novel bromodomain and extra-terminal domain inhibitors (BETi) were identified by screening a DNA-encoded chemical library (DEL) provided by WuXi AppTec (Shanghai, China) using the BD1 and BD2 domains of BRD2, BRD3, and BRD4 proteins. Proteins were generated by amplifying the relevant gene sections encoding BRD2, BRD3, and BRD4, followed by their cloning into pET28a vectors (Novagen, Northumberland, UK). These constructs were transformed into *Escherichia coli* BL21(DE3) cells (Novagen), and protein expression was induced through incubating with 0.2 mM isopropyl β-D-thiogalactopyranoside (IPTG) for 16 h at 18 °C. Bacterial cells were harvested, resuspended in lysis buffer (50 mM Tris, pH 8.2, Thermo Fisher Scientific, Waltham, MA, USA; 300 mM NaCl, LPS Solution, Daejeon, Republic of Korea; and 20 mM imidazole, Sigma-Aldrich, St. Louis, MO, USA), and then sonicated. The lysates were centrifuged at 1550× *g* for 1 h at 4 °C, and the supernatant was loaded onto a HisTrap HP nickel affinity column (GE Healthcare, Chicago, IL, USA). The column was washed, and BRD2, BRD3, and BRD4 were eluted using 50 mM Tris buffer (pH 8.2) containing 300 mM NaCl and 500 mM imidazole.

### 2.2. Time-Resolved Fluorescence Resonance Energy Transfer (TR-FRET) Assay

TR-FRET assay kits specific for BRD2 (BD1 + BD2), BRD3 (BD1 + BD2), and BRD4 (BD1 + BD2) from BPS Bioscience (San Diego, CA, USA) were utilized. Master mixtures containing 1× BRD homogeneous assay buffer and diluted BD ligands were prepared. BRD proteins were thawed on ice and diluted using 1× BRD homogeneous assay buffer. Subsequently, 1.5 μL of the master mix was added to each well of a microplate (Labcyte, San Francisco, CA, USA), and reactions were initiated by adding 5 μL of diluted BRD protein to each well. The plates were then incubated at 20–22 °C for 30–60 min. GSH acceptor beads (Perkin Elmer, Waltham, MA, USA) and streptavidin-conjugated donor beads (Perkin Elmer, Waltham, MA, USA) were diluted in a 1× BRD homogeneous detection buffer. Next, 10 μL of the acceptor bead mixture was added to each well. After a 30 min incubation at 18 °C, 10 μL of the donor bead mixture was added to each well, followed by incubation for 15–30 min at 18 °C. Alpha particle counts were determined using an EnVision 2105 multimode plate reader (Perkin Elmer, Waltham, MA, USA).

### 2.3. Binding Kinetics Assay

Binding kinetics analysis was performed using the Sartorius Octet^®^ R2 system (Sartorius, Göttinge, Germany). Streptavidin biosensors were loaded with biotinylated BRD2 BD1, BRD2 BD2, BRD3 BD1, BRD4 BD1, BRD4 BD2, or biotin, which was used as a control in phosphate-buffered saline with Tween 20 (PBST) buffer [PBS (LPS solution), 0.02% Tween 20 (LPS solution), and 5% dimethyl sulfoxide (DMSO)]. The sensors were then immersed in a compound solution for 10 min. The binding of compounds to the proteins was assessed by dipping individual sensors into a series of protein dilutions for 180 s to measure the association rate (k_on_), and the dissociation rate (k_off_) was measured in blank PBST buffer. A parallel control sensor was dipped in blank PBST. Competitive binding assays were performed by immersing the compound sensor in a mixture of proteins and the test compounds.

### 2.4. Mouse Primary Chondrocytes and Reagents Used for Treatment

ICR mice (4-day-old; DBL, Eumseong-kun, Republic of Korea) were euthanized and disinfected with 70% ethanol. Using sterile tools, the skin around each mouse’s knee was removed, and the knee joints were carefully dissected. The knee joints were washed with sterile PBS, incubated for 2–3 h at 37 °C, and digested using collagenase type II and trypsin-EDTA in Dulbecco’s Modified Eagle Medium. Cartilage was collected in a new Petri dish and incubated with collagenase type II in DMEM for 2 h at 37 °C. The filtered solution was centrifuged, and chondrocyte pellets were collected. For in vitro experiments, chondrocytes were seeded in DMEM (10% fetal bovine serum) in 35 mm culture dishes. After approximately three days, the cells were co-treated with BBC0906 (at concentrations of 5, 10, and 20 µM) and interleukin (IL)-1β (1 ng/mL) (Piscataway, NJ, USA) for 12 h [25]. The animal experiments conducted at Sungkyunkwan University were approved by the University’s Institutional Animal Care and Use Committee.

### 2.5. Lactate Dehydrogenase (LDH) Assay to Assess Cell Viability

Chondrocytes seeded on 35 mm culture plates were incubated for approximately four days, and BBC0906 was added to the culture plates at various concentrations, followed by incubation for three days. The culture media were collected and analyzed using an LDH kit (BioVision, Milpitas, CA, USA). Phosphate-buffered saline (PBS) was used as a negative control, corresponding to 100% viability, and Triton X-100 was used as a positive control, corresponding to 0% viability. The optical density of each group was measured at 490 nm using a SYNERGY H1 microplate reader (BioTek, Winooski, VT, USA) at 490 nm [26]. The cell viability was calculated as follows:

(1) $$Cell\;Viability\;\%=100-\frac{sample-negative\;control}{positive\;control-negative\;control}\times 100,$$

### 2.6. Reverse Transcription-Polymerase Chain Reaction (RT-PCR) and Quantitative (Q)RT-PCR

TRIzol (Molecular Research Center, Cincinnati, OH, USA) was used to isolate the total RNA from the articular chondrocytes. The RNA was reverse-transcribed into cDNA using ImProm-II Reverse Transcriptase (Promega, Madison, WI, USA). SYBR Premix Ex Taq (TaKaRa Bio Inc., Shiga, Japan) was used for PCR amplification. Real-time PCR (qRT-PCR) was performed, and the gene transcript levels were normalized to that of GAPDH [27]. The following primers were used: mouse SOD1 (5′-AACCAGTTGTGTTGTCAGGAC-3′ and 5′-CCACCATGTTTCTTAGAGTGAGG-3′); mouse SOD2 (5′-AGGAGAGTTGCTGGAGGCTA-3′ and 5′-TAGTAAGCGTGCTCCCACAC-3′); mouse CAT (5′-AGCGACCAGATGAAGCAGTG-3′ and 5′- TCCGCTCTCTGTCAAAGTGTG-3′); mouse GPX1 (5′-AGTCCACCGTGTATGCCTTCT-3′ and 5′-GAGACGCGACATTCTCAATGA-3′); and mouse GAPDH (5′-TCACTGCCACCCAGAAGAC-3′ and 5′-TGTAGGCCATGAGGTCCAC-3′).

### 2.7. Protein Isolation and Western Blotting

To extract proteins from chondrocytes, a lysis buffer (150 mM NaCl, 1% NP-40, 50 mM Tris, 0.2% sodium dodecyl sulfate, and 5 mM NaF; Roche, Basel, Switzerland) was supplemented with protease and phosphatase inhibitors. MMP3 and MMP13 in the conditioned medium were precipitated using trichloroacetic acid (TCA). To remove TCA, the protein pellet was centrifuged after treatment with acetone. Antibodies against MMP3 (Abcam, Cambridge, UK), MMP13 (Abcam), COX2 (Abcam), ERK1/2, pERK, c-Jun N-terminal kinase (JNK), pJNK, p38, and pp38 (Cell Signaling Technology, Danvers, MA, USA) were used for Western blot analyses. Anti-ERK1/2 antibodies were used to detect protein levels. The ERK activity-corresponding band intensities were adjusted by performing the densitometric analysis (AlphaEase FC 4.0; Alpha Innotech, San Lendro, CA, USA) [27,28].

### 2.8. Human OA Cartilage and Experimental OA in Mice

Human OA cartilage obtained from patients undergoing knee arthroplasty was used as the undamaged and damaged human cartilage (Supplementary Table S1). All patients provided written informed consent, and the collection was approved (UC14CNSIO150). Cutting the medial meniscal tibial ligament during DMM surgery destabilizes the medial meniscus. Male C57BL/6 mice (12 weeks old) underwent DMM surgery according to a previously published protocol [29]. The intra-articular (IA) injection of BBC0906 was conducted once weekly for seven weeks (beginning three weeks after surgery) to treat experimental OA in DMM mice. The mice were examined again 10 weeks after DMM surgery. As previously described [29], histological analysis was performed using safranin O staining. The knee joints of the dead mice were fixed in paraformaldehyde and decalcified for two weeks using EDTA. An ethanol/xylene gradient was used to dehydrate the paraffin blocks and cast the knee joints. Serial microtome sections (5-µm-thick) were obtained from the paraffin blocks. The sections were hydrated via deparaffinization and xylene substitution, and an ethanol gradient was performed before safranin O staining. Stained knee joint slices were examined for cartilage degradation using the Osteoarthritis Research Society International (OARSI) grading system and by assessing the subchondral bone plate thickness. The OARSI grading system is typically used to measure OA severity from grades 0 to 6 [25]. Grade 0 indicates intact cartilage morphology, whereas grades 1–6 suggest an aberrant matrix, superficial zone cells, surface discontinuities, vertical fissures, erosion, denudation, and deformation. Immunohistochemistry was performed to examine cartilage protein expression. The hydration process resembled that of safranin O staining. We used an antigen-retrieval reagent, background blocking reagent, primary antibodies against each protein [MMP3 (Abcam, Cambridge, UK), MMP13 (Abcam), and COX2 (ProteinTech Group, Rosemont, IL, USA)], and secondary antibodies against each primary antibody. Finally, aminoethyl carbazole detection was performed to visualize the samples before quantification using the ImageJ 1.54i software (National Institutes of Health, Bethesda, MD, USA).

### 2.9. High-Performance Liquid Chromatography (HPLC) Analysis of BBC0906

BBC0906 was analyzed using high-performance liquid chromatography (HPLC) using an HPLC system (Shimadzu LC-20AD; Shiseido, Japan). The column system was composed of an analytical column (Kinetex EVO C18 4.6 × 50 mm, 5 µm, Phenomenex, Torrance, CA, USA). The photodiode array (PDA) detector was set at 220 and 254 nm. The mobile phase consisted of 10% ACN (0.02% TFA) in water (0.04% TFA) and 80% ACN in water. The gradient elution was eluted within 3.00 min. The flow rate was initially set to 1.5 mL/min, followed by a hold at 80% ACN for 0.70 min. The flow rate was again set to 1.5 mL/min, returning to 10% ACN in water, followed by holding for 0.30 min. Finally, the flow rate was set at 2.0 mL/min.

### 2.10. Statistical Analysis

Each experiment was independently repeated at least four times. To analyze three or more groups, the statistical significance was assessed using one-way analysis of variance (ANOVA) and Tukey’s post hoc test for normally distributed data, and both a Kruskal–Wallis and Dunn’s post hoc test were used for nonparametric data. The Mann–Whitney U test was used to analyze the two groups for nonparametric data. The results are presented as the mean ± SEM. GraphPad Prism 9 (GraphPad Software, San Diego, CA, USA) was used for all analyses, and the significance was set at *p* < 0.05.

## 3. Results

### 3.1. BBC0906, Which Targets the BD2 Domain of BET Proteins, Was Produced

**Figure 1:**
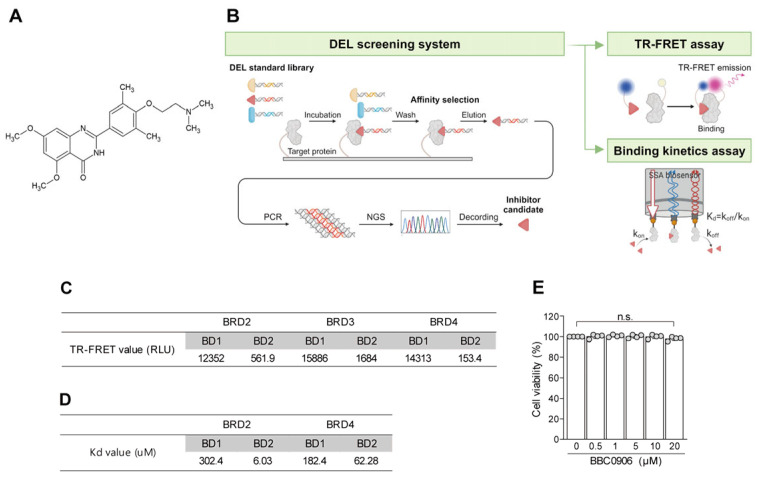
BBC0906 is a BET inhibitor targeting the BD2 domain. (**A**) Chemical structure of BBC0906. (**B**) The image shows the general process by which BBC0906 was selected as a BET inhibitor. BBC0906 was selected using the DEL screening system and examined using TR-FRET and binding kinetics assays. (**C**,**D**) Each table shows the TR-FRET value (**C**) and Kd value (**D**) of BBC0906 obtained through TR-FRET and binding kinetics assays. (**E**) Cytotoxicity of BBC0906 to chondrocytes was evaluated using an LDH assay. Data are represented as the mean ± SEM as results of one-way ANOVA with Tukey’s post hoc test (*n* = 4) (**E**). Differences from the PBS (control) group are denoted as n.s. = not significant.

BBC0906 (Figure 1A) was selected using a DEL screening system (Figure 1B). This system contains chemical compounds that bind to the target proteins. Each chemical has a DNA sequence that acts as a name tag, and when several chemicals are poured onto the desired target protein and washed away, only chemicals that can bind to the protein remain. This system was used to analyze the DNA sequence attached to a chemical to identify the corresponding chemical. BBC0906, which was selected using this screening method, was synthesized, and its purity was confirmed via HPLC (Supplementary Figure S1). The binding of BBC0906 to the BD2 domain of BET proteins was verified using TR-FRET and binding kinetics assays. In particular, when comparing the binding force of BBC0906 to the BD1 domain of each BET protein, a high binding affinity for the BD2 domain of BET proteins was observed (Figure 1C,D). The cytotoxicity of BBC0906 in primary chondrocytes was also evaluated (Figure 1E). The chondrocytes were incubated with BBC0906 at various concentrations (0.5, 1, 5, 10, and 20 µM) for three days. According to the LDH assay results, BBC0906 at the selected concentration range did not affect the viability of chondrocytes.

### 3.2. BBC0906 Suppressed ROS Production and Catabolic Expression through NOX4

**Figure 2:**
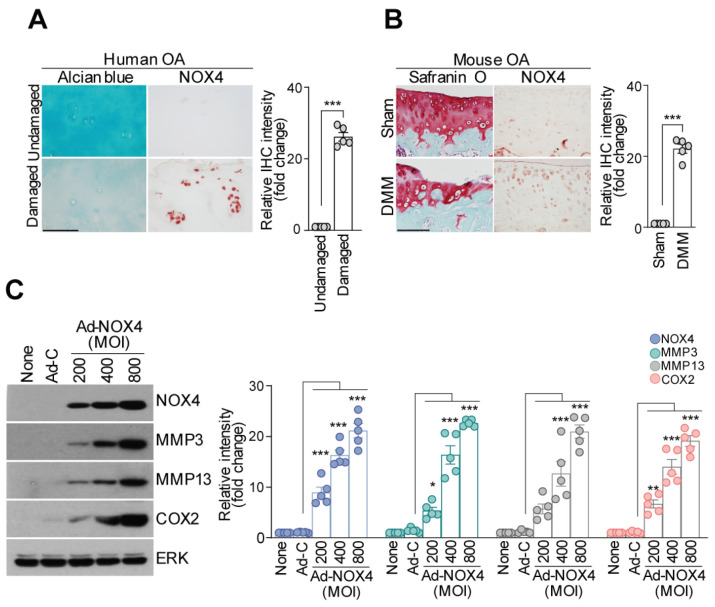
NOX4 upregulation in cartilage induces OA pathogenesis. (**A**) NOX4 protein levels in damaged and undamaged samples (**left panel**) were compared, and NOX4 protein levels in undamaged and damaged human knee joint cartilage were compared using IHC (scale bar = 100 μm) (**right panel**). (**B**) Ten weeks after DMM surgery was performed on C57BL/6 mice, the extent of cartilage degradation was assessed using safranin O staining (**left panel**). Additionally, NOX4 protein expression level in the cartilage was determined using IHC staining (scale bar = 100 μm) (**right panel**). (**C**) Mouse chondrocytes were infected with the NOX4 adenovirus. The expression levels of MMP3, MMP13, and COX2 were confirmed using Western blotting (**left panel**). The expression levels of each protein were normalized to those of ERK and are presented as relative values among the groups (**right panel**). Data are presented as the mean ± SEM as results of the analysis using the Mann–Whitney U test (**A**,**B**) and one-way ANOVA with Tukey’s post hoc test (*n* = 5) (**C**). Significant differences from the PBS (control) group are denoted as * *p* < 0.05, ** *p* < 0.01, and *** *p* < 0.001.

Nicotinamide adenine dinucleotide phosphate oxidase 4 (NOX4) is a member of the NOX family of NADPH oxidases and an important endogenous source of ROS [30]. NOX4-induced ROS production causes oxidative stress and injury to chondrocytes [31,32,33]. The NOX4 protein levels were higher in damaged human knee joint cartilage than in undamaged cartilage (Figure 2A). We used DMM surgery to create a mouse OA model and analyzed mouse knee joint cartilage 10 weeks after DMM surgery. When OA was induced, the expression of NOX4 was higher in DMM mice than in WT mice (Figure 2B). When mouse chondrocytes were infected with Ad-NOX4, which overexpresses NOX4, the expression of MMP3, MMP13, and COX2, which are factors that damage cartilage, increased (Figure 2C). In addition, NOX4-knockout mice, which do not express NOX4, did not show cartilage damage, whereas WT mice did, even when DMM surgery was performed, and the expression of MMP3, MMP13, and COX2, which damage mouse cartilage, was reduced. Since NOX4 expression was suppressed, the expression of the ROS-related factor 8-OHdG, a downstream molecule, was reduced in NOX4-knockout DMM mice (Supplementary Figure S2A–C). As previously reported [34], NOX4 affects OA, and its activation induces an environment that is prone to OA development.

ROS can cause OA. Chondrocytes suffer from oxidative stress in the presence of ROS, resulting in the increased secretion of catabolic factors that metabolize cartilage [35]. In Duchenne muscular dystrophy, ROS levels can be regulated by the pharmacological inhibition of the BET protein, alleviating disease symptoms [36].

**Figure 3:**
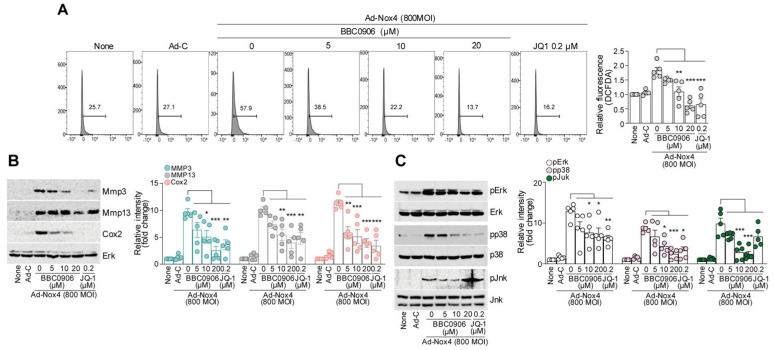
BBC0906 suppresses ROS production, catabolic factor expression, and mitogen-activated protein kinase (MAPK) signaling. (**A**) The cellular DCFDA assay was performed on mouse articular chondrocytes. NOX4 overexpressing chondrocytes were treated with BBC0906 at concentrations ranging from 0 to 20 μM. ROS activity in live cells is presented as histograms for quantitative assessment in live cell samples (**left panel**) and as bar graphs for relative assessment (**right panel**). (**B**) Chondrocytes overexpressing NOX4 were treated with BBC0906, and the expression of MMP3, MMP13, and COX2 was examined using Western blotting (**left panel**) and through determining the relative protein intensity levels (**right panel**). (**C**) In chondrocytes overexpressing NOX4, the regulation of MAPK signaling by BBC0906 was examined using Western blotting (**left panel**), and the relative intensity of each phosphorylated protein between the groups was determined (**right panel**). Data are presented as mean ± SEM as the result of a one-way ANOVA with Tukey’s post hoc test (*n* = 5). Significant differences from the PBS (control) group are denoted as * *p* < 0.05, ** *p* < 0.01, and *** *p* < 0.001.

Therefore, we verified the effectiveness of the newly discovered potential BET inhibitor, BBC0906, in chondrocytes under oxidative stress. The overexpression of NOX4 in chondrocytes can induce oxidative stress by increasing ROS levels [37]. Intracellular ROS levels decreased as BBC0906 concentration increased and were analyzed using fluorescence-activated cell sorting (Figure 3A). Oxidative stress increases when the balance between prooxidants and antioxidants is disrupted, and we examined the changes in antioxidant enzymes. The major antioxidant enzymes include superoxide dismutase (SOD), catalase (CAT), and glutathione peroxidase (GPx). We confirmed that, among antioxidant enzymes, SOD1 and SOD2, but not CAT and GPX1, were restored in expression as the concentration of BBC0906 increased (Supplementary Figure S3).

In addition, treatment with BBC0906 reduced the expression of catabolic factors that were increased by NOX4 (Figure 3B). In OA, catabolic factors are primarily regulated by MAPK signaling. Therefore, we investigated the effect of BBC0906 on MAPK signaling and found that MAPK signaling, which was activated by NOX4, was decreased by BBC0906 (Figure 3C). This reduction occurred because BBC0906 suppressed pathways downstream of NOX4. For example, BBC0906 suppresses MAPK signaling, which is activated by NOX4 overexpression in mouse articular chondrocytes.

In addition, we used JQ1 as a comparative inhibitor of BBC0906. JQ1 is a bromodomain inhibitor that selectively binds to the aminoterminal pair bromodomain of BET proteins and inhibits their ability to read acetylated lysine residues [38,39]. JQ1 decreased the production of ROS and expression of catabolic factors (Figure 3A,B). BBC0906 inhibited the JNK, ERK, and p38 pathways, whereas JQ1 failed to inhibit the JNK pathway (Figure 3C). Moreover, JQ1 did not regulate antioxidant enzymes (Supplementary Figure S3).

### 3.3. BBC0906 Intra-Articular Injection Suppressed the Degradation of Mouse Articular Cartilage

**Figure 4:**
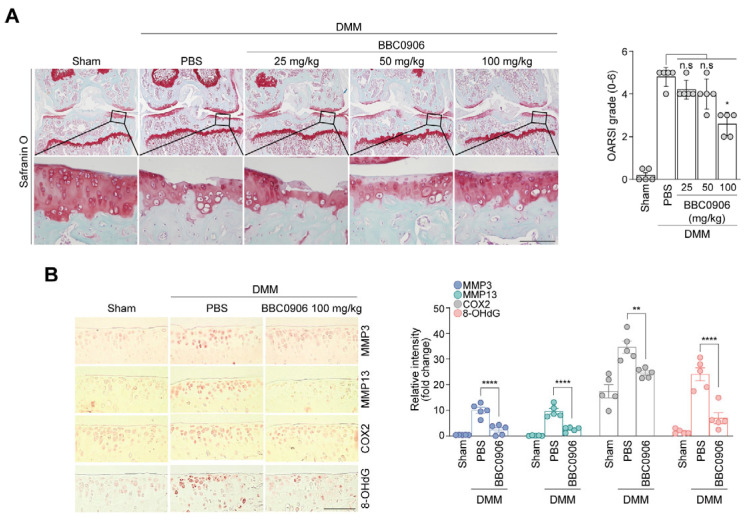
BBC0906 suppressed cartilage degradation and ROS production and alleviated OA. (**A**,**B**) C57BL/6 male mice (12 weeks old) underwent DMM surgery, received intra-articular injections of BBC0906 once a week, and were analyzed after being euthanized 10 weeks after surgery. For histological analysis, safranin O staining was conducted (scale bar = 100 μm) ((**A**), **left panel**), and OA severity was evaluated using the OARSI grade ((**A**), **right panel**). The protein levels of the catabolic factors and ROS were obtained using IHC staining ((**B**), **left panel**) and were analyzed in each sham group ((**B**), **right panel**). Data are presented as the mean ± SEM as results of the one-way ANOVA with Tukey’s post hoc test (*n* = 5) (**A**) and Kruskal–Wallis with Dunn’s post hoc test (*n* = 5) (**B**). Significant differences from the PBS (control) group are denoted as follows: * *p* < 0.05, ** *p* < 0.01, **** *p* < 0.0001, and n.s = not significant.

We established an OA mouse model to confirm the OA inhibitory effects of BBC0906 in mice. The OA mouse model was created using the DMM surgery described above, and BBC0906 was IA injected into the knee joints of mice starting from the fourth week post-surgery. The progression of OA was confirmed by performing safranin O staining 10 weeks after the surgery, and OA was alleviated as the BBC0906 concentration increased. The OARSI grade values, which indicate the severity of OA, were significantly lower in the groups injected with BBC0906 than in those injected with PBS (Figure 4A).

IHC experiments conducted on mouse cartilage revealed that BBC0906 reduced the expression of catabolic factors that degraded cartilage and decreased the levels of 8-OHdG. This suggests that BBC0906 prevented cartilage degradation and reduced oxidative stress levels within the cartilage (Figure 4B). Previous reports and our in vitro results suggest that MAPK activation is associated with OA [40]; therefore, we investigated the association of MAPK signaling pathways with in vivo OA models. The results showed that the MAPK signaling pathway (ERK, p38, and JNK) was increased in the DMM model, while a reduction in the intra-articular injections of BBC0906 was observed in the DMM model (Supplementary Figure S4). As expected, these results indicated that BBC0906 is involved in OA progression by regulating MAPK signaling.

## 4. Discussion

OA is a degenerative joint disease characterized by cartilage breakdown that usually affects the hands, knees, hips, and spine, leading to pain, stiffness, and decreased mobility [41,42,43]. The disease progresses as the protective cartilage that cushions the ends of bones wears down over time, causing the bones to rub against each other. This can lead to inflammation and a further breakdown of joint tissue [5].

We used two systems to mimic OA conditions and showed their effectiveness: BBC0906 in NOX4-overexpressing chondrocytes and the DMM surgery mouse model. The role of NOX4 in OA pathogenesis has received increasing attention because of its pivotal role in ROS production [31]. NOX4, a member of the NADPH oxidase family, is a major source of ROS in various cell types, including chondrocytes [44,45,46]. NOX4 overexpression in OA cartilage leads to excessive ROS generation, which contributes to oxidative stress and subsequent cartilage degradation [37].

ROS, including superoxide anions and hydrogen peroxide, are critical mediators of oxidative stress and have been implicated in the pathophysiology of OA. Elevated ROS levels can induce chondrocyte apoptosis, MMP activation, and the degradation of extracellular matrix components, such as collagen and aggrecan, which are essential for maintaining cartilage integrity [47,48,49,50]. ROS production is affected by antioxidant enzymes such as superoxide dismutase (SOD), catalase, and glutathione peroxidase. Antioxidant enzymes suppress cell damage and oxidative stress by neutralizing ROS, which is important for the pathogenesis of OA [48,51,52]. In this study, BBC0906 increased the expression of SOD1 and SOD2, which can affect ROS generation or scavenge free radicals, but JQ1 was not involved in the change in the expression of antioxidant enzymes. BBC0906 restored the expression of antioxidant enzymes disrupted by the overexpression of NOX4.

In human endothelial cells, NOX4 induces ROS production and activates MAPK signaling [53]. The MAPK pathway is a representative signaling pathway in OA that plays a central role in the expression of catabolic factors in OA [34,54,55]. The JNK, ERK, and p38 signaling pathways are involved in the MAPK pathway.

When these signaling pathways are activated in chondrocytes, MMPs are upregulated, apoptosis is promoted, chondrocyte proliferation is inhibited, and OA progresses [56,57,58]. NOX4 increases ROS production and causes oxidative stress in chondrocytes. The MAPK pathway is also activated, which increases the expression of catabolic factors. OA can be induced under these conditions.

When NOX4-overexpressing chondrocytes were treated with BBC0906, we observed a reduction in MAPK signaling and ROS levels. Consequently, we confirmed that the induction of OA by NOX4 was inhibited by suppressing the expression of catabolic factors.

The other system utilized was the DMM model, a commonly used OA animal model [59,60]. The DMM model induces OA by destabilizing the meniscus through the transection of the ligaments connecting the meniscus, leading to cartilage degradation [61]. Following DMM surgery, the intra-articular injection of BBC0906 alleviated OA; thus, BBC0906 exhibited therapeutic effects in the most commonly used mouse model of OA.

BET inhibitors include selective and pan-inhibitors. Recently, selective BET inhibitors have been compared with pan-BET inhibitors. For example, ABBV-744, a BD2 selective inhibitor discovered after ABBV-075, inhibited the growth of specific cell lines, unlike ABBV-075, which shows widespread cell growth inhibition. ABBV-744 also had a lower effect on overall transcription than ABBV-075 did. This selective inhibition shows promise for solving disease problems without disrupting normal physiological activities [62]. However, according to the patent, BET inhibitors specific or selective for BD2 prevented diastolic dysfunction, but ABBV-744 elevated diastolic dysfunction which was likely due to its on-target inhibition of the androgen receptor [63].

In our study, BBC0906 showed effects on chondrocytes within 12 h, but no toxicity was observed, even after three days. This demonstrated the low cytotoxicity of BBC0906. However, because the potential side effects in vivo have not been investigated, there are limitations to the toxicity assessment. Therefore, future in vivo toxicity assessments, including biodistribution and clearance studies, as well as specific organ toxicity tests, are necessary to confirm these findings. If these studies yield positive results, BBC0906 could be a promising candidate for the treatment of OA.

## 5. Conclusions

**Figure 5:**
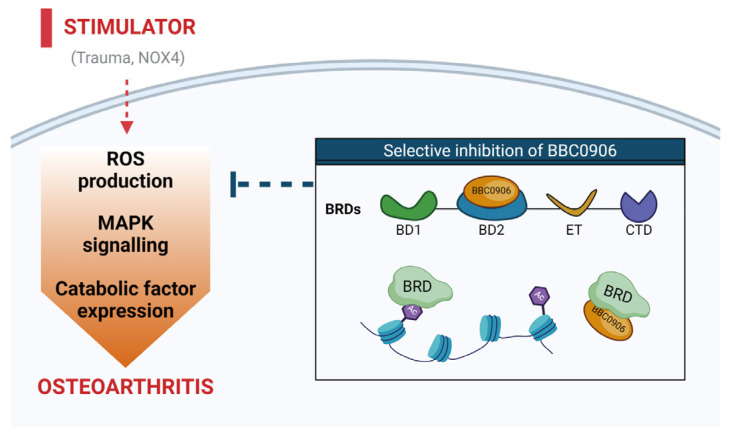
Graphic summary of the mechanism underlying the effect of BBC0906 in OA pathogenesis. ROS, reactive oxygen species; MAPK, mitogen-activated protein kinase.

In this study, we developed BBC0906, which can specifically bind to the BD2 domain of BET proteins through DEL screening. This compound regulates oxidative stress in chondrocytes and modulates MAPK signaling, which is a key pathway in OA. BBC0906 reduces the expression of catabolic factors, thereby inhibiting cartilage degradation. Therefore, BBC0906 is a potential osteoarthritic treatment agent that preserves normal physiological activities by selectively inhibiting the BD2 domain of BET proteins (Figure 5).

## Data Availability

The original contributions presented in the study are included in the article/Supplementary Material; further inquiries can be directed to the corresponding authors.

## Supplementary Materials

The following supporting information can be downloaded from https://www.mdpi.com/article/10.3390/antiox13080943/s1, Supplementary Figure S1: BBC0906 was subjected to HPLC using a Shimadzu LC-20AD system; Supplementary Figure S2: Nox4 regulates ROS and catabolic factors in OA pathogenesis; Supplementary Figure S3: Expression levels of antioxidant enzymes in chondrocytes treated with Ad-NOX4 and BBC0906; Supplementary Figure S4: BBC0906 suppresses the activation of MAPK signaling in OA pathogenesis; Table S1: Characteristics of the human specimens used in this study.
