# Supplementary material for: A Novel BD2-Selective Inhibitor of BRDs Mitigates ROS Production and OA Pathogenesis
Source: Antioxidants (Basel). 2024 Aug 2;13(8):943. doi: 10.3390/antiox13080943 (PMC11352053; doi:10.3390/antiox13080943)
Supplement: Supplementary file 1 [file antioxidants-13-00943-s001.zip › antioxidants-3078491-supplementary.pdf]

Table S1 Characteristics of the human specimens used in this study.

No	Age (years) /gender	ICRS grade	Joint	Height (m)	Weight (Kg)	BMI (kg/m^2)	Use
1	72/F	4	Knee	165	65	23.88	IHC
2	63/F	4	Knee	152	52	22.51	IHC
3	69/F	4	Knee	151	60	26.31	IHC
4	73/F	4	Knee	153.	70.75	29.89	IHC
5	73/F	4	Knee	154	83	35	IHC

Figure S1

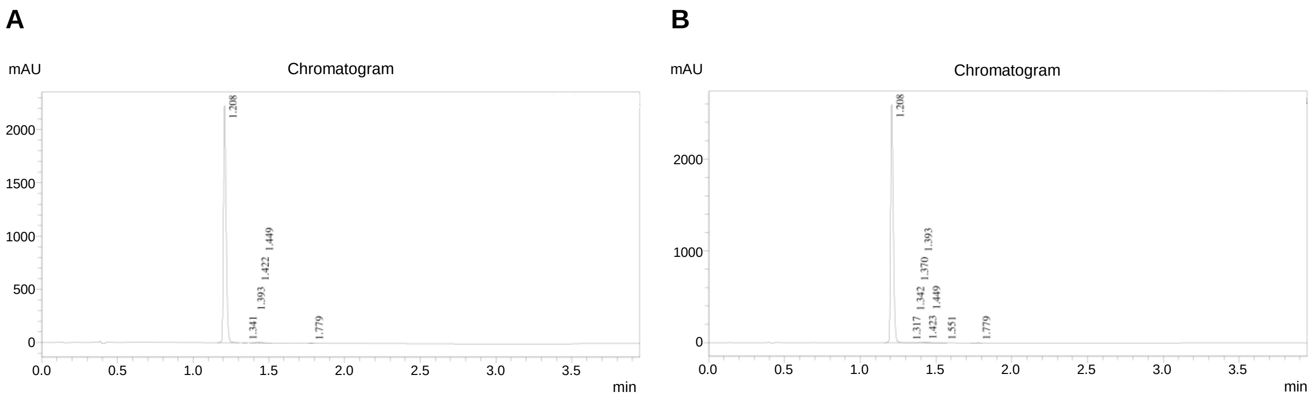

Supplementary Figure S1. BBC0906 was subjected to HPLC using a Shimadzu LC-20AD system. (A, B) BBC0906 was analyzed using a PDA detector at 220 nm (A) and 254 nm (B).

Figure S2

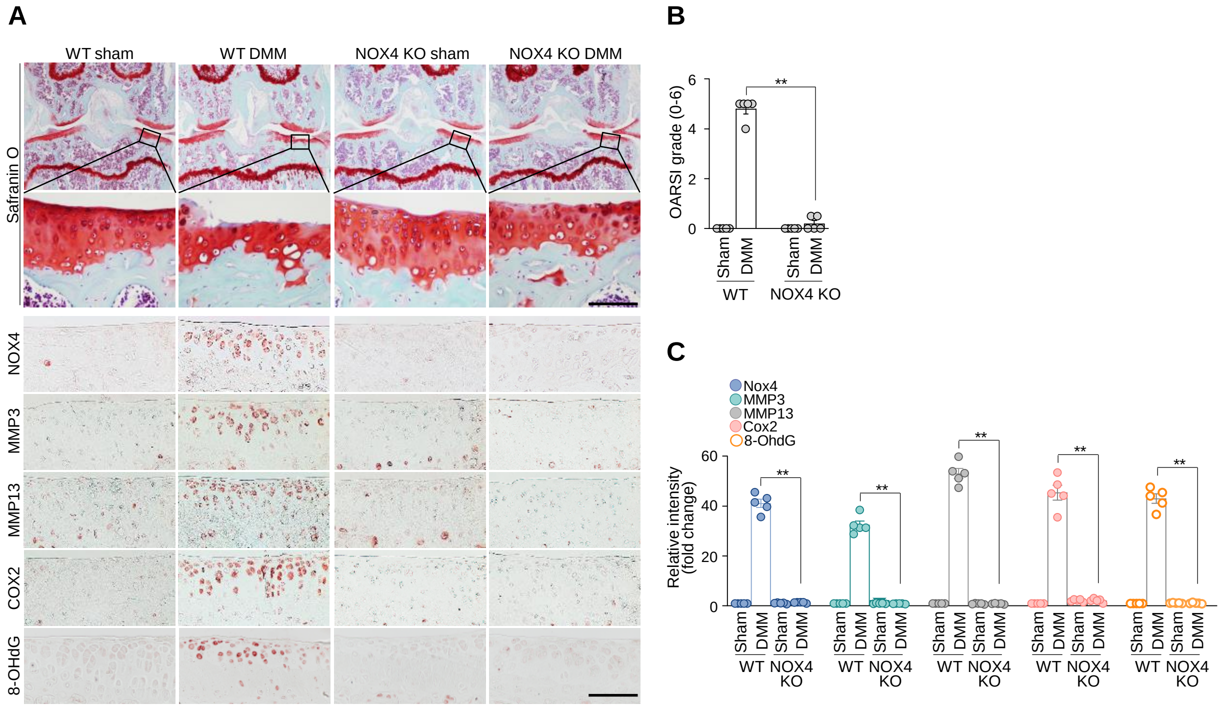

Supplementary Figure S2. Nox4 regulates ROS and catabolic factors in OA pathogenesis. (A-C) After DMM surgery, cartilage degradation and protein levels were evaluated in Nox4 knockout mice and compared with WT mice (n = 5). Cartilage degradation was analyzed using safranin O staining (A), and the severity of each group was scored using the OARSI grade (B). The degree of protein expression in each group is expressed as the relative intensity of IHC staining based on WT sham (C). The Mann–Whitney U test was used for nonparametric data. Results are presented as the mean ± SEM. Scale bar = 100 μm ** $p < 0.01$.

Figure S3

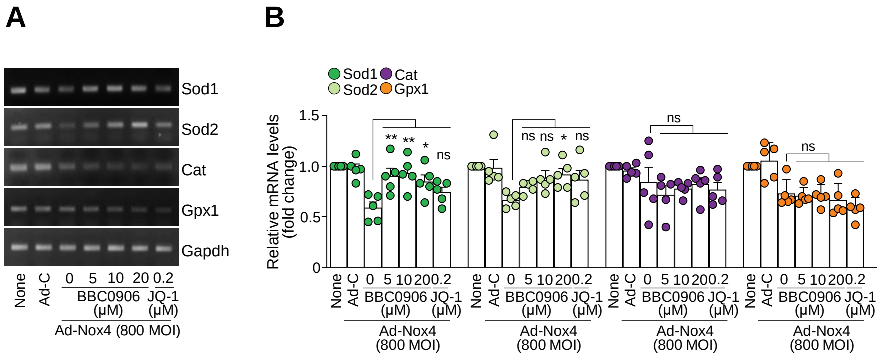

Supplementary Figure S3. Expression levels of antioxidant enzymes in chondrocytes treated with Ad-NOX4 and BBC0906. Representative RT-PCR images (A) and qRT-PCR analysis (B) of antiox-idant enzymes in chondrocytes infected with Ad-NOX4 in the absence or presence of BBC0906. Data are presented as mean ± SEM as the result of a one-way ANOVA with Tukey's post-hoc test (n = 5). Significant differences from the control group are denoted as *P < 0.05, **P < 0.01, and ns = not significant.

Figure S4

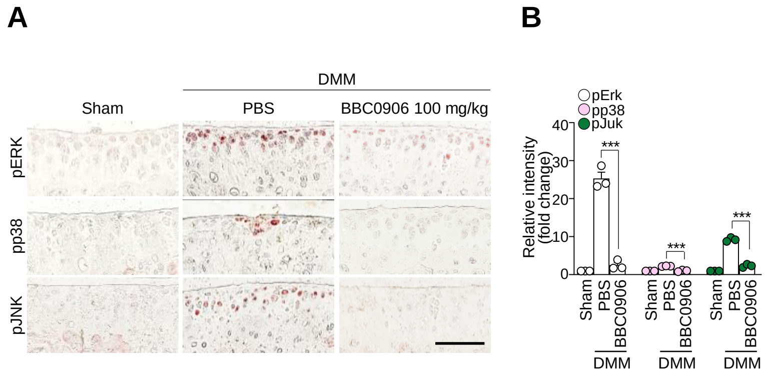

Supplementary Figure S4. BBC0906 suppresses the activation of MAPK signaling in OA patho-genesis. Representative immunostaining images (A) and immunostaining intensity of each pro-tein expression (B) of DMM-operated mice that received intra-articular injections of BBC0906 and were sacrificed at the indicated weeks after surgery (n = 3). Data are presented as mean ± SEM as the result of a one-way ANOVA with Tukey's post-hoc test (n = 3). Significant differences from the PBS (control) group are denoted as ***$P < 0.001$. Scale bar = 100 μm.
